# Isotopic Fractionation Associated With Sulfate Import and Activation by *Desulfovibrio vulgaris* str. Hildenborough

**DOI:** 10.3389/fmicb.2020.529317

**Published:** 2020-09-18

**Authors:** Derek A. Smith, David A. Fike, David T. Johnston, Alexander S. Bradley

**Affiliations:** ^1^Department of Earth and Planetary Sciences, Washington University in St. Louis, St. Louis, MO, United States; ^2^Department of Earth and Planetary Sciences, Harvard University, Cambridge, MA, United States; ^3^Division of Biology and Biomedical Sciences, Washington University in St. Louis, St. Louis, MO, United States

**Keywords:** enzymes, sulfur, oxygen, isotope fractionation, chemostat, sulfate reduction, sulfate permease, sulfate adenylyl transferase

## Abstract

The use of stable isotopes to trace biogeochemical sulfur cycling relies on an understanding of how isotopic fractionation is imposed by metabolic networks. We investigated the effects of the first two enzymatic steps in the dissimilatory sulfate reduction (DSR) network – sulfate permease and sulfate adenylyl transferase (Sat) – on the sulfur and oxygen isotopic composition of residual sulfate. Mutant strains of *Desulfovibrio vulgaris* str. Hildenborough (DvH) with perturbed expression of these enzymes were grown in batch culture, with a subset grown in continuous culture, to examine the impact of these enzymatic steps on growth rate, cell specific sulfate reduction rate and isotopic fractionations in comparison to the wild type strain. Deletion of several permease genes resulted in only small (∼1‰) changes in sulfur isotope fractionation, a difference that approaches the uncertainties of the measurement. Mutants that perturb Sat expression show higher fractionations than the wild type strain. This increase probably relates to an increased material flux between sulfate and APS, allowing an increase in the expressed fractionation of rate-limiting APS reductase. This work illustrates that flux through the initial steps of the DSR pathway can affect the fractionation imposed by the overall pathway, even though these steps are themselves likely to impose only small fractionations.

## Introduction

Dissimilatory sulfate reduction (DSR) is associated with the oxidation of organic matter in modern and ancient oceans, and is essential to regulating the redox state of the Earth’s surface. The sulfur cycle represents one of the oldest biologically mediated chemical cycles on earth (Shen et al., 2001; Canfield, 2004; Fike et al., 2015). The marine sulfur cycle can be tracked through time by examining the stable isotopic compositions of seawater sulfate and reduced sedimentary sulfur phases, such as pyrite or organic sulfur. Sulfur and oxygen stable isotope ratios in sulfate and other sulfur-bearing species are largely a product of the biogeochemical sulfur cycle, although they can also be affected by abiotic processes like hydrothermal activity and photochemical reactions in the atmosphere. DSR is particularly influential on the sulfur isotope ratios (Jørgensen, 1982; Canfield, 2001; Brunner et al., 2005; Canfield et al., 2010; Sim et al., 2011a, 2019; Leavitt et al., 2013) because sulfur isotopic fractionations (^34^ε_sulfate–sulfide_) generated by DSR can be very large – with observations recording and models predicting values ranging up to 72‰ (Habicht and Canfield, 1997; Wortmann et al., 2001; Brunner and Bernasconi, 2005; and as reported in Sim et al., 2011a). However, the actual fractionations imposed during DSR are usually smaller, and depend on a number of environmental and biological factors such as sulfate reduction rate, sulfate concentration, temperature and the fluxes and fractionations imposed by the particular set of enzymes in a given organism (Thode et al., 1951; Rudnicki et al., 2001; Farquhar et al., 2003; Bradley et al., 2011, 2016; Sim et al., 2011b; Leavitt et al., 2013). Therefore, an essential approach to understanding the fractionations involves defining the fluxes of sulfur molecules through the DSR pathway, the fractionations of isotopes at each enzymatic step, and the controls on these in various organisms.

**FIGURE 1:**
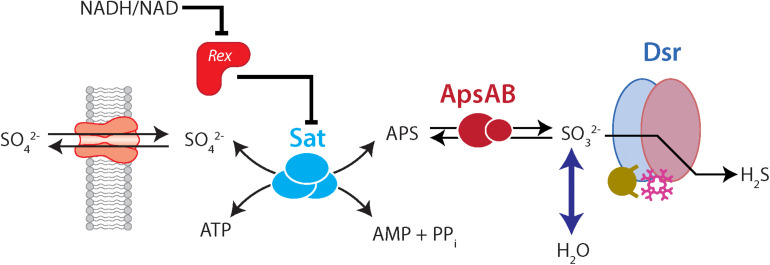
The dissimilatory sulfate reduction pathway, indicating the role of Rex in regulating Sat and isotopic fractionation. Forward reactions are left-to-right, while right-to-left indicates reversal. In the forward (reductive) direction, sulfate enters the cell through transporters. Sat catalyzes the reaction of sulfate with ATP to the products APS, AMP, and pyrophosphate (PP_*i*_). APS is reduced to sulfite, and sulfite is reduced to sulfide via a complex interaction with Dsr. Exchange of oxygen isotopes happens between sulfite and water. Rex represses Sat expression. However, when the cellular redox state (as expressed by NADH/NAD) is high, Rex un-represses Sat, and Sat is more highly expressed. This would allow more flux through Sat. In our mutant strains this is likely to result in a higher intracellular APS concentration, which may allow for more expression of fractionation by ApsR.

In sulfate-reducing bacteria (SRB) carrying out DSR, four critical enzymatically catalyzed steps (Figure 1) are involved: (i) import of sulfate into the cell by sulfate transporters, (ii) the “activation” of sulfate to the intermediate adenylyl 5′-phosphosulfate (APS) by sulfate adenylyl transferase (Sat, EC 2.7.7.4, also called ATP sulfurylase), (iii) the reduction of APS to sulfite by adenylyl-sulfate reductase (ApsR, EC 1.8.99.2, also called APS reductase), and (iv) the reduction of sulfite to sulfide (and potentially to thionates) by the multi-subunit dissimilatory sulfite reductase (Dsr, EC 1.8.99.5) (Brunner and Bernasconi, 2005; Wing and Halevy, 2011; Leavitt et al., 2015; Santos et al., 2015; Bradley et al., 2016; Sim et al., 2019).

The sulfur isotope ratio of residual sulfate produced by DSR is a consequence of the fractionation imposed by the enzymes in DSR. However, isotopic fractionations expressed by individual steps are not well constrained. The intrinsic sulfur isotope effects for two enzymes of the DSR metabolic network have recently been measured *in vitro*. Under experimental conditions, DsrAB imposed a fractionation factor (^34^ε_reactant–product_) of 15.3 ± 2‰ (Leavitt et al., 2015) and ApsR had a ^34^ε of 20.1 ± 0.8‰ (Sim et al., 2019). However, these enzymatic constraints cannot fully explain the bank of observations.

The stable isotope ratio of oxygen in residual sulfate during DSR is a consequence of fractionation (^18^ε) resulting from both reductive and oxidative reactions (Brunner et al., 2005; Farquhar et al., 2008; Turchyn et al., 2010; Wankel et al., 2014). Sulfate does not exchange oxygen isotopes with water at biologically relevant pH and temperature ranges (Chiba and Sakai, 1985; Brunner et al., 2005). However, DSR promotes an increase in the relative amount of ^18^O in residual sulfate through two mechanisms: (i) discrimination against the heavier isotope during sulfate reduction (Brunner et al., 2005; Antler et al., 2013), and (ii) equilibrium exchange between water and sulfite (Betts and Voss, 1970; Farquhar et al., 2008; Wankel et al., 2014). Back-reactions from sulfite to sulfate can carry this equilibrium signature into sulfate (Lloyd, 1968; Fritz et al., 1989; Brunner et al., 2005; Turchyn et al., 2010; Antler et al., 2013; Wankel et al., 2014).

The isotopic consequences of the first steps in DSR – sulfate import and activation – have not been closely examined. These steps are unlikely to have large direct sulfur isotope effects, since they are not associated with forming or breaking bonds to sulfate sulfur. However, they may still affect the overall sulfur isotope fractionation during DSR by perturbing flux through the metabolic network. In this study, we begin to examine these effects by comparing a model sulfate reducing organism [*Desulfovibrio vulgaris* str. Hildenborough (DvH)] to mutant strains in which these early steps of sulfate reduction have been perturbed.

The import of sulfate into the cytoplasm of cells is modulated by sulfate permeases. The specificity and affinity of these transporters is not well documented (e.g., Price et al., 2018). Many SRB possess multiple sulfate permeases, and some data hint that differences in permeases among strains may have isotopic consequences. For example, *Desulfovibrio alaskensis* str. G20 and DvH show very different ^34^ε under identical growth conditions under low millimolar sulfate concentrations (Bradley et al., 2016). One interpretation of this observation is that these strains are adapted to different concentrations of ambient sulfate – with *D. vulgaris* having high-affinity sulfate transporters adapted to low sulfate conditions, and *D. alaskensis* having low-affinity sulfate transporters adapted to high sulfate conditions. According to this interpretation, under low sulfate conditions *D. vulgaris* would have a higher intracellular sulfate concentration and therefore express higher ^34^ε. *D. alaskensis* has eleven annotated sulfate transporters (Kuehl et al., 2014), and DvH has three (Clark et al., 2006). However, a recent study showed that across 23 different bacterial genera 48% of ABC transport proteins were incorrectly annotated or failed to accurately describe the transported substrates (Price et al., 2018). The genomes of 44 sulfate-reducing archaea and bacteria were recently examined for putative sulfate transporters and provide important targets for future research directions (Marietou et al., 2018). This analysis, published after our experiments were conducted and analyzed, revealed that instead of three putative sulfate transporters, DvH actually possesses thirteen transporter type proteins that could be associated with sulfate reduction (Marietou et al., 2018). Under conditions in which a strain is perturbed such that the only altered variable is the sulfate transport rate (i.e., a permease mutant with an unchanged growth phenotype), we would predict more quantitative reduction of imported sulfate, and lower ^18^ε and ^34^ε. Under real-world experimental conditions, we need to account for complexity resulting from other changes to growth.

To study the importance of sulfate transport, we employed a mutant strain (ΔPerm) of DvH in which three proteins previously annotated as sulfate transport proteins (gene loci DVU0053, DVU0279, and DVU1999) were deleted from the genome (Keller et al., 2009). This triple-knockout mutant was predicted to be deficient in sulfate transport relative to the wild type, although it is still capable of sulfate reduction. We compared ΔPerm to the wild type (WT) strain and to three strains in which ΔPerm was complemented with each individual permease (DVU0053, DVU0279, and DVU1999). Of these three genes, DVU0053, closely aligns phylogenetically with other known sulfate transporters in the SulP family of proteins (Marietou et al., 2018), while DVU0279 is most closely related to dicarboxylic acid transporters, and DVU1999 is associated with bicarbonate transporters (Marietou et al., 2018). Of the remaining ten putative sulfate transporters in DvH, many are likely involved in phosphate transportation, three are putative sulfite transporters, and only one other (DVU2958) aligns with highly conserved sulfate transporters (Marietou et al., 2018).

After transport into the cytoplasm, sulfate is converted to adenosine 5′-phosphosulfate (APS) by Sat. This requires an investment of cellular energy in the form of ATP. Cells optimize the production of APS by modulating Sat expression through a redox-sensitive transcriptional repressor, Rex (Ravcheev et al., 2012; Christensen et al., 2015). Rex modulates transcription in response to the intracellular ratio of NADH/NAD^+^ through attachment to a DNA binding site upstream of its target gene (Gyan et al., 2006; Kuehl et al., 2014). This Sat expression optimization may be an adaptation to minimize APS hydrolysis, which would expend ATP investment. Perturbation of this regulatory feedback may result in increased intracellular APS production. This could result in an increased intracellular APS pool, which could result in a larger fractionation being expressed during APS reduction to sulfite (and an overall larger cellular ^18^ε and ^34^ε). A larger pool of intracellular APS or higher rates of Sat expression could also result in more back-reaction from sulfite to sulfate.

To study the consequences of Rex on isotopic fractionation, we compared a wild type DvH to a mutant strain lacking Rex (Δ*rex*) and to a mutant strain lacking the Rex binding site (IR1and2) (Christensen et al., 2015). The Δ*rex* strain cannot transcriptionally repress Sat with Rex, and Sat transcripts in this strain are ∼11 times more abundant than in the wild type strain under growth conditions similar to our experiments (Christensen et al., 2015). However, Rex may interact with other regions of the genome, and so the phenotype of this strain may be more complicated than the perturbation to Sat expression alone (Christensen et al., 2015). The IR1and2 strain perturbs the Rex binding site and still contains Rex, but since Rex cannot bind to DNA upstream of Sat it cannot transcriptionally repress Sat expression. This mutant specifically impairs the mechanism for Sat repression, and shows a mean transcript level for Sat ∼4.6 higher than wild type (Christensen et al., 2015). We examined the ^18^ε and ^34^ε of these mutant strains relative to the wild type strain.

## Materials and Methods

### Strains and Batch Growth Conditions

Seven strains of *D. vulgaris*: WT, ΔPerm, Perm53, Perm279, Perm1999, Δ*rex*, and IR1and2, were obtained from J. D. Wall (University of Missouri) (Table 1). The construction of the mutants has been previously reported (Keller et al., 2009; Korte et al., 2014; Christensen et al., 2015). All seven strains were grown in triplicate batch culture at 30°C in the dark. Each seventy-milliliter butyl rubber sealed serum bottle contained 60 mL of MO media (as defined in Zane et al., 2010) without added yeast extract, of which 5 mL was derived from the inoculum. The remaining volume was a headspace of oxygen-free nitrogen gas. Fresh media contained 25 mM lactate as the electron donor and 28 mM sulfate as the electron acceptor. A 97.6% H_2_^18^O (Berry & Associates/ICON Isotopes) spike was added to the media water of the Rex-related mutants (Δ*rex* and IR1and 2) and a WT to achieve a δ^18^O_H__2__O_ of +130‰ vs. VSMOW and permit the profiling of back reactions through the DSR network. The bottles were autoclaved after the addition of the ^18^O-enriched water. Sterile and anaerobic protocols and conditions were maintained throughout growth and sampling. Inocula were grown under the same conditions as the experimental strains, but without the ^18^O spike.

**TABLE 1:** Strains of *Desulfovibrio vulgaris* str. Hildenborough used in this study.

Strain	Strain	Description	Source or References
WT	JW710	Strain that all mutants in this study were created from	Keller et al., 2009
IR1and2	JW9320	Mutant lacking Rex binding site in promotor region of Sat encoding gene	Christensen et al., 2015
Δ*rex*	JW3319	Knockout mutant of gene (*rex*) that encodes for Rex	Korte et al., 2014
ΔPerm	JW9201	Triple knockout of the 3 genes that were annotated to encode for sulfate permeases	J. D. Wall Laboratory
Perm1999	JW9357	JW9201 with complemented gene DVU1999, an annotated sulfate permease	J. D. Wall Laboratory
Perm53	JW9355	JW9201 with complemented gene DVU0053, an annotated sulfate permease	J. D. Wall Laboratory
Perm279	JW9356	JW9201 with complemented gene DVU0279, an annotated sulfate permease	J. D. Wall Laboratory

### Batch Sampling

The experimental matrix contained 144 experimental bottles (36 WT and 18 of each mutant strain) and 17 killed controls, with 60 ml of culture in each bottle. After inoculation of each strain, three sample bottles were each harvested at 6 time points.

Harvested material was divided into two portions. At the time of harvest, a 15 mL aliquot was removed from each bottle, from which a 0.5 mL sub-sample was used to measure optical density at 600 nm using a Thermo Nanodrop 2000c. The remaining 14.5 mL were centrifuged at 5000 rpm for 15 min at 5°C. The supernatant was decanted and stored at −20°C. The cell pellet was re-suspended in 0.75 mL 10 mM PBS buffer, transferred to a microcentrifuge tube, and centrifuged at 10,000 rpm for 10 min at room temperature. The cell pellet was stored at −20°C and the PBS buffer was discarded.

To the remaining 45 mL of culture in the sealed serum bottle, we injected 4 mL of 1 M (sulfate free) ZnCl_2_ using a needle and syringe. This mixture was shaken vigorously, then allowed to react for a minimum of 10 min in order to ensure that both gaseous and aqueous sulfide was converted to ZnS. The butyl septum and aluminum seal were then removed. Two 1.5 mL aliquots were removed for the measurement of dissolved ion concentrations (see below). The remaining 46 mL of media and ZnCl_2_ were centrifuged at 5000 rpm for 15 min at 5°C. The ZnS pellet was retained and the supernatant was transferred to a new tube. The ZnS pellets were each rinsed three times with 45 mL MilliQ water, homogenized, centrifuged at 5000 rpm for 15 min at 5°C, then the supernatant was discarded. The washed ZnS pellet was re-suspended in 25 mL MilliQ water and reacted with 4 mL 2M AgNO_3_ for more than 96 h in the dark, in order to completely convert the ZnS to Ag_2_S. The supernatant remaining from the ZnS precipitation was allowed to react with 3.5 mL 1M BaCl_2_ for more than 96 h in the dark in order to ensure complete conversion of aqueous sulfate into the insoluble BaSO_4_.

Ag_2_S and BaSO_4_ solutions were each centrifuged at 5000 rpm for 10 min at 5°C. The supernatants were discarded. The pellets were then homogenized three times in 45 mL of MilliQ water and centrifuged again at 5000 rpm for 10 min at 5°C, with the supernatant being discarded after each centrifugation. The Ag_2_S pellet was then homogenized and reacted with 15 mL 1 M NH_4_OH. The solution was centrifuged at 5000 rpm for 10 min at 5°C. The supernatant was discarded and the resultant pellet was twice rinsed with 45 mL of MilliQ water, followed by centrifugation at 5000 rpm for 10 min at 5°C. The prepared BaSO_4_ and Ag_2_S pellets were dried in an oven at 70°C overnight, homogenized, and weighed for isotopic analysis.

### Batch Rate Estimations

In batch culture, we calculated cellular growth rates and cell-specific sulfate reduction rate (csSRR). Growth rates for each strain were calculated from the slopes of the linear regression of the natural logarithm of the optical densities (OD_600_) of the cells growing in exponential phase versus time. The csSRR up to time of harvest was calculated using the concentrations of sulfate and the OD_600_ at time of harvest. The average OD_600_ over the exponential growth period was taken as

$$\mathrm{Average}\mathrm{optical}\mathrm{density}=\frac{c_{t}}{kt\left[1-e^{kt}\right]}$$

where c_t_ is the optical density at harvest, *k* is the specific growth rate, and *t* is the time elapsed since inoculation. OD_600_ was converted to cell number using a calibration previously established for *D. vulgaris* (Leavitt et al., 2013). Sulfate consumption per cell was converted to mean csSRR in units of fmol sulfate/cell/day.

### Chemostat Design and Growth Conditions

**FIGURE 2:**
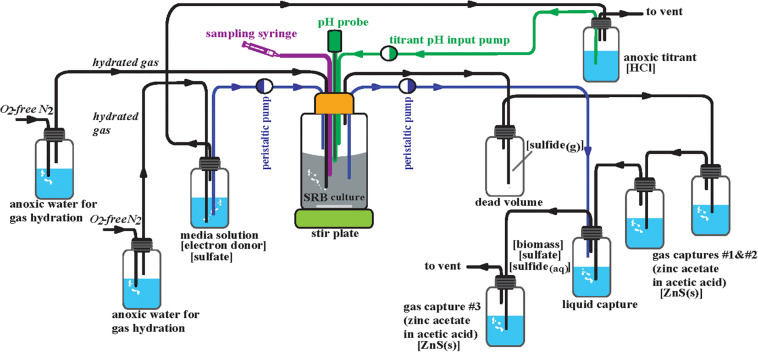
Schematic design for the chemostats.

The WT and Δ*rex* strains were cultivated in separate anoxic continuous cultivation reactors run in parallel under chemically static conditions (chemostats) (Figure 2). The initial design of these is described in Leavitt et al. (2013, 2016). MO media (described above) for each strain were prepared under anoxic conditions (10 L per strain), autoclaved, kept under sterile conditions, and kept anoxic by a constant flux of 0.22 μm filter sterile ultra high purity (>99.999%) anoxic N_2_ gas at an influent pressure of 2.5 psi. This gas stream was used maintain anoxic conditions of the 1 M HCl titrant via constant flushing of the titrant headspace. The growth chambers (reactors) were maintained at a working volume of 300 mL. Prior to inoculation the reactors were autoclaved with 300 mL of MO media. Sterile and anoxic conditions were maintained throughout the course of the experiment. A constant stream of 0.22 μm filter sterile ultra high purity (>99.999%) anoxic N_2_ gas was supplied to each reactor at an influent pressure of 6.5 psi that flowed in series to all of the gas and liquid traps. Inoculation occurred for each strain from a 10 mL aliquot of batch culture in MO media. These inocula were grown from freezer stock then transferred three times and harvested at exponential phase. Each reactor was connected to a series of three gas traps, each containing 50 mL of 1 M zinc acetate to precipitate ZnS: two following the dead volume for evolved H_2_S gas from the reactor and one following the liquid capture of spent media and cells from the reactor, insuring quantitative conversion of ZnS (Figure 2). Each reactor was also connected to a liquid trap where spent media and cells accumulated containing excess 1 M BaCl_2_ at a pH of 4 which immediately halts biologic activity and precipitates BaSO_4_. Sulfide was maintained well below detection limits (<30 μM, see below) in this liquid trap via constant gas purging and connection in tandem to the third zinc trap. The pH of the reactors was maintained at 7.15 ± 0.4. An Etatron DLX pH-RX/MBB metering pump, activated by a pH probe, supplied anoxic and sterile 1 M HCl for titration of each reactor. Dilution rate was altered by adjusting the pump rate of an Ismatec SC0816 peristaltic pump containing 0.64 mm two-stop PVC Tygon tubing. Five steady-state dilution rates were examined: 0.008 ± 0.0002, 0.011 ± 0.0006, 0.015 ± 0.0005, 0.022 ± 0.0004, and 0.051 ± 0.0024 hr^–1^. In chemostats at steady state, the growth rate (μ) is equal to the dilution rate. These dilution rates relative to μ_max_ were 0.096 ± 0.002, 0.133 ± 0.008, 0.181 ± 0.004, 0.265 ± 0.002, and 0.614 ± 0.027 for the WT and 0.129 ± 0.003, 0.177 ± 0.010, 0.242 ± 0.005, 0.355 ± 0.003, and 0.823 ± 0.035 for Δ*rex* (Table 2). The onset of steady state was defined by constant cell density, as monitored by the optical density at 600 nm (OD_600_) of the reactor. Once steady state was achieved, a minimum of three flush volumes [the volume required to replace all of the liquid in the reactor] occurred before sampling commenced. After these three flush volumes, the gas and liquid traps were switched with new traps containing fresh material to start steady state sample collection.

**TABLE 2:** Average growth rate, cell-specific sulfate reduction rate (csSRR), and isotopic fractionation factors for the wild type (WT and WT_2_), Rex-promoter Sat mutant (IR1and2), Rex mutant (Δ*rex*), triple permease knockout mutant (ΔPerm), and the three mutants with an individual permease complemented back into the triple knockout (Perm1999, Perm53, and Perm279) displayed with errors in measurements or uncertainties (±).

ID	Growth Type	Growth rate (μmax, hr^–1^)	Dilution rate (relative to μmax)	Avg csSRR (fmol/cell/day)	^18^ε_*SO4*_ (‰)	^34^ε_*SO4*_ (‰)	^34^Δ_*SO4*_ (‰)
WT_1_	batch	0.083 ± 0.007	NA	117.6 ± 54.4	15.4 ± 1.9	7.1 ± 0.6	NM
IR1and2	batch	0.061 ± 0.003	NA	110.0 ± 26.8	23.8 ± 1.3	11.8 ± 0.5	NM
Δ*rex*	batch	0.062 ± 0.004	NA	50.2 ± 59.3	29.5 ± 2.5	12.3 ± 0.8	NM
WT_2_	batch	0.112 ± 0.014	NA	73.9 ± 46.7	NM	6.8 ± 0.4	NM
ΔPerm	batch	0.074 ± 0.011	NA	105.0 ± 28.5	NM	7.9 ± 0.7	NM
Perm1999	batch	0.082 ± 0.004	NA	92.4 ± 37.3	NM	8.1 ± 0.4	NM
Perm53	batch	0.135 ± 0.021	NA	NM	NM	NM	NM
Perm279	batch	0.140 ± 0.020	NA	NM	NM	NM	NM
WT_1_	continuous	0.008 ± 0.0002	0.096 ± 0.002	13.1 ± 1.2	NM	23.4 ± 0.2	22.7 ± 0.1
WT_1_	continuous	0.011 ± 0.0006	0.133 ± 0.008	13.4 ± 1.3	NM	24.5 ± 0.2	22.8 ± 0.1
WT_1_	continuous	0.015 ± 0.0003	0.181 ± 0.004	17.6 ± 1.7	NM	20.8 ± 0.1	20.3 ± 0.1
WT_1_	continuous	0.022 ± 0.0002	0.265 ± 0.002	22.3 ± 2.2	NM	21.3 ± 0.2	21.0 ± 0.1
WT_1_	continuous	0.051 ± 0.0022	0.614 ± 0.027	45.6 ± 4.4	NM	13.0 ± 0.1	12.5 ± 0.1
Δ*rex*	continuous	0.008 ± 0.0002	0.129 ± 0.003	16.0 ± 1.6	NM	29.8 ± 0.2	29.6 ± 0.1
Δ*rex*	continuous	0.011 ± 0.0006	0.177 ± 0.010	14.8 ± 1.4	NM	24.1 ± 0.2	23.7 ± 0.1
Δ*rex*	continuous	0.015 ± 0.0003	0.242 ± 0.005	17.2 ± 1.7	NM	24.4 ± 0.1	24.0 ± 0.1
Δ*rex*	continuous	0.022 ± 0.0002	0.355 ± 0.003	27.7 ± 2.7	NM	22.6 ± 0.2	23.4 ± 0.1
Δ*rex*	continuous	0.051 ± 0.0022	0.823 ± 0.035	66.3 ± 6.4	NM	18.2 ± 0.1	17.9 ± 0.1

*Those parameters not measured are listed as NM, and those not applicable are listed as NA. All fractionation factors (ε and Δ) are determined as reactant – product and the subscript refers to the chemical species for which the value is being reported.*

### Chemostat Sampling

Steady-state sampling included samples directly from the reactor, gas and liquid traps. Sampling from all three locations provided an assessment of isotopic consistency, shown in Table 2. A 20 mL aliquot was taken from the reactor, placed in two 15 mL centrifuge tubes, and centrifuged at 5,000 rpm for 10 min at 4°C. The resulting cell pellet was resuspended with 0.75 mL 10 mM PBS in microcentrifuge tubes, centrifuged at 12,000 rpm for 6 min, the supernatant discarded and pellet frozen at −20°C. The supernatant of each 10 mL sample was reacted with 2.5 mL 1 M ZnCl_2_ for 25 min and centrifuged at 5,000 rpm for 10 min at 4°C. The resultant ZnS was retained for later isotope analysis. A 1.5 mL aliquot was taken from the supernatant of the centrifuged reactor sample for IC analysis (described below) and stored at −20°C. The remaining liquid was reacted with 1.5 mL of 1 M BaCl_2_ and allowed to react for at least 24 h, then centrifuged at 5,000 rpm for 10 min at 4°C. The resultant BaSO_4_ was retained for isotope analysis. The accumulated precipitated ZnS was harvested from the gas traps in three 50 mL centrifuge tubes, and centrifuged at 5,000 rpm for 10 min at 4°C. The ZnS pellets were combined, rinsed 3x with MilliQ water then reacted with excess 1 M AgNO_3_ to generate Ag_2_S and allowed to react for at least 24 h in the dark. These Ag_2_S pellets were washed 3x with MilliQ water with homogenization and centrifugation in between the washing steps. The washed Ag_2_S was then reacted with 15 mL of 1 M ammonium hydroxide, homogenized and centrifuged, then washed 3x with MilliQ water and dried at 70°C overnight. The precipitated BaSO_4_ from the liquid trap was collected in three or four 50 mL centrifuge tubes [dependent on accumulated volume] and centrifuged at 5,000 rpm for 10 min at 4°C. The pellets were combined, rinsed with MilliQ water three times, and homogenized. The resulting pellets were purified using the diethylenetriamine pentaacetate (DTPA) method of Bao (2006) then dried at 70°C overnight.

### Chemostat Rate Estimations

In chemostats, csSRR was determined in a manner similar to batch experiments. OD_600_ was converted to cell number (Leavitt et al., 2013), and moles of sulfate consumed was determined by the difference at sampling from initial individual media batches.

### Dissolved Ions

Aliquots of media designated for analysis of dissolved ion concentrations were centrifuged at 10,000 rpm for 10 min at room temperature and the supernatant was transferred to a new microcentrifuge tube and frozen at −20°C until analysis. Samples were diluted in MilliQ water (1: 50) for analysis by ion chromatography (IC). Sulfate, lactate and acetate concentrations were determined using suppressed anion chromatography with conductivity detection (Metrohm 881 Compact IC Pro equipped with a Metrosep A Supp 7-250/4.0 column and a 2 mM Na_2_CO_3_ eluent or A Supp 5-100/4.0 column and mix of 1 mM Na_2_CO_3_, 3.2 mM NaHCO_3_ with 25 mL HPLC grade acetone per L eluent). Sulfate, lactate, and acetate had detection limits better than 150, 150, and 800 μM, respectively. Sulfide concentrations were determined by diluting samples in MilliQ water (1: 65) and then performing spectrophotometric analysis following the methods of Cline (1969). The detection limits were 30 μM.

### Isotope-Ratio Mass Spectrometry (IRMS)

Sulfur isotope ratios were measured in duplicate on each sample of precipitated BaSO_4_ and Ag_2_S from each experiment. Approximately 0.4 mg of material with 2 mg of V_2_O_5_ were loaded in tin capsules and analyzed on an Isoprime 100 IRMS interfaced with an Elementar vario ISOTOPE cube elemental analyzer (for the Rex mutant set) (Virginia Tech), or Thermo Scientific Finnigan Delta V Plus IRMS interface with a Costech 4010 elemental combustion system (Permease mutant set) (Washington University). All sulfur isotope values are reported as per mil variations relative to VCDT following the conventional delta notation:

δ34S=((S34/32S)Sample(S34/32S)VCDT-1).

Values of δ^34^S were calibrated relative to international standards of BaSO_4_ (IAEA-SO-5) and AgS_2_ (IAEA-S-1 and IAEA-S-3). The external precision of **δ**^34^S is ±0.25‰ (1σ) as estimated from repeated, long-term measurements of internal and international reference materials.

Oxygen isotope ratios were measured on duplicate samples of BaSO_4_ from each experiment. Approximately 0.4 mg of material was loaded in silver capsules and analyzed using a Thermo Finnigan high temperature conversion elemental analyzer (TC/EA) coupled to a Thermo Delta Plus IRMS. All oxygen values are reported as per mil variations relative to VSMOW following conventional delta notation. Values of δ^18^O were calibrated relative to international standards of BaSO_4_ (GSW-1, NBS-127, IAEA-SO-5, IAEA-SO-6). The external precision of δ^18^O is ≤0.2‰ (1σ) as estimated from repeated, long-term measurements of internal and international reference materials.

The sulfur and oxygen isotopic fractionation factors were determined following conventional methods. For the batch experiments [closed system], the apparent fractionation factors for a given set of experiments were calculated from linear regressions of δ values against the negative natural logarithm of the fraction of the reactant remaining (Hayes, 2001)

$$\delta_{measured}=\delta_{initial}-\text{ln}\left(f\right)*\varepsilon$$

where *f* is the fraction of reactant remaining and ε is the fractionation factor. Uncertainty in the regressions is reported as the 95% confidence interval around a best-fit slope. Regressions were compared to each other using analysis of variance (ANOVA) to test for homogeneity of the regression slopes between pairs of regressions. For the chemostat experiments [open system], the isotopic fractionation factor was calculated from the difference between the initial and out flowing reactant divided the fraction of the product produced (Hayes, 1983, 2001)

$$\varepsilon =-\left(\frac{\delta_{measured}-\delta_{initial}}{f_{product}}\right)$$

where *f* is the fraction of product produced (sulfide) and ε is the fractionation factor of sulfate. All ε are determined as reactant – product and the subscript refers to the chemical species for which the value is being reported.

## Results

### Growth Characteristics and Reaction Stoichiometry

**FIGURE 3:**
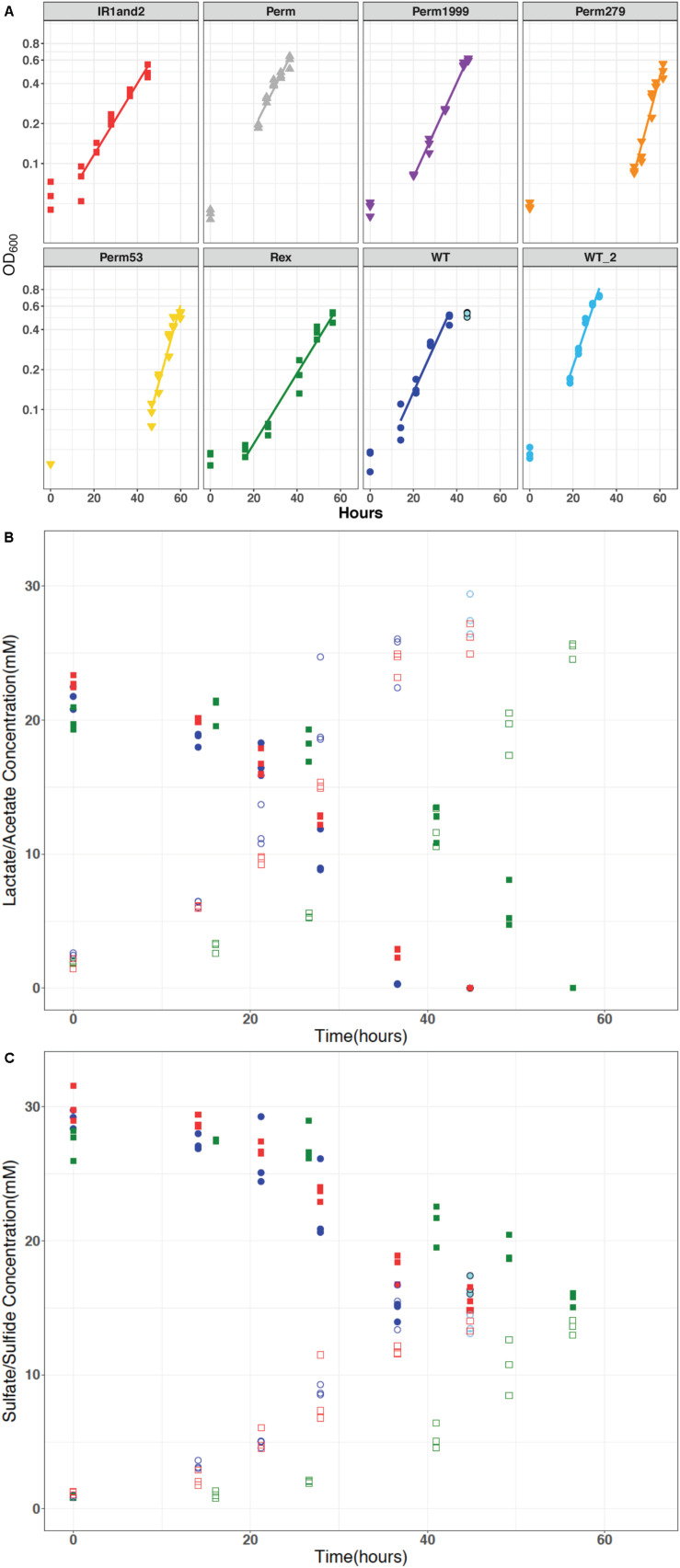
**(A)** Optical density measured at 600 nm (log scale) plotted against time for all DvH strains: wild type (WT and WT_2_), triple permease knockout (ΔPerm), and the three individually expressed permeases: Perm53, Perm279, and Perm1999, Rex-promoter Sat mutant (IR1and2), and Rex mutant (Δ*rex*), which show similar growth characteristics. **(B)** The representative concentrations of lactate and acetate versus time. These concentrations changed relative to sulfate as predicted by equation 1. **(C)** Representative concentrations of sulfate and sulfide for all strains were similar, varied inversely throughout the experiment as expected, and maintained mass-balance. The data for WT, IR1and2, Δ*rex* are in dark blue, red, and green, respectively. In all panels, the stationary phase time points for the wild type are indicated in light blue.

Permease mutants were able to grow as sulfate reducers, suggesting that the annotated transport proteins are not the sole mechanism for sulfate import into DvH cells (Figure 3A). The Δ*rex* mutant exhibited a longer lag phase and grew more slowly than the WT strain (Figure 3A). Strain IR1and2 showed growth characteristics similar to Δ*rex* (Figure 3A). All isotopic results reported here derive from samples collected from exponential phase growth, with the exception of the final time point for the WT strain. The stationary phase timepoints are omitted from our analyses due to the differences in stress response, gene expression, translation, transcription, cell morphology and physiology induced during stationary phase (e.g., Kolter et al., 1993; Clark et al., 2006; Navarro Llorens et al., 2010). Growth rates and cell-specific sulfate-reduction rates (csSRRs) are shown in Table 2.

All the strains showed similar reactant consumption rates and reaction stoichiometry. The governing stoichiometry for sulfate reduction via the oxidation of lactate (Rabus et al., 2013):

$2{\text{CH}}_{3}{\text{CHOHCOO}}^{-}+{\text{SO}}_{4}^{2-}↔2{\text{CH}}_{3}{\text{COO}}^{-}+2{\text{HCO}}_{3}^{-}+{\text{HS}}^{-}+{\text{H}}^{+}$ held for all strains (Figures 3B,C). Differences in organic acid consumption and production were not observed across the strains. The similar dissimilatory consumption and production rates but differing growth rates likely implies an efficiency difference among the mutants.

### Sulfur and Oxygen Isotope Fractionations Change With Batch Growth

**FIGURE 4:**
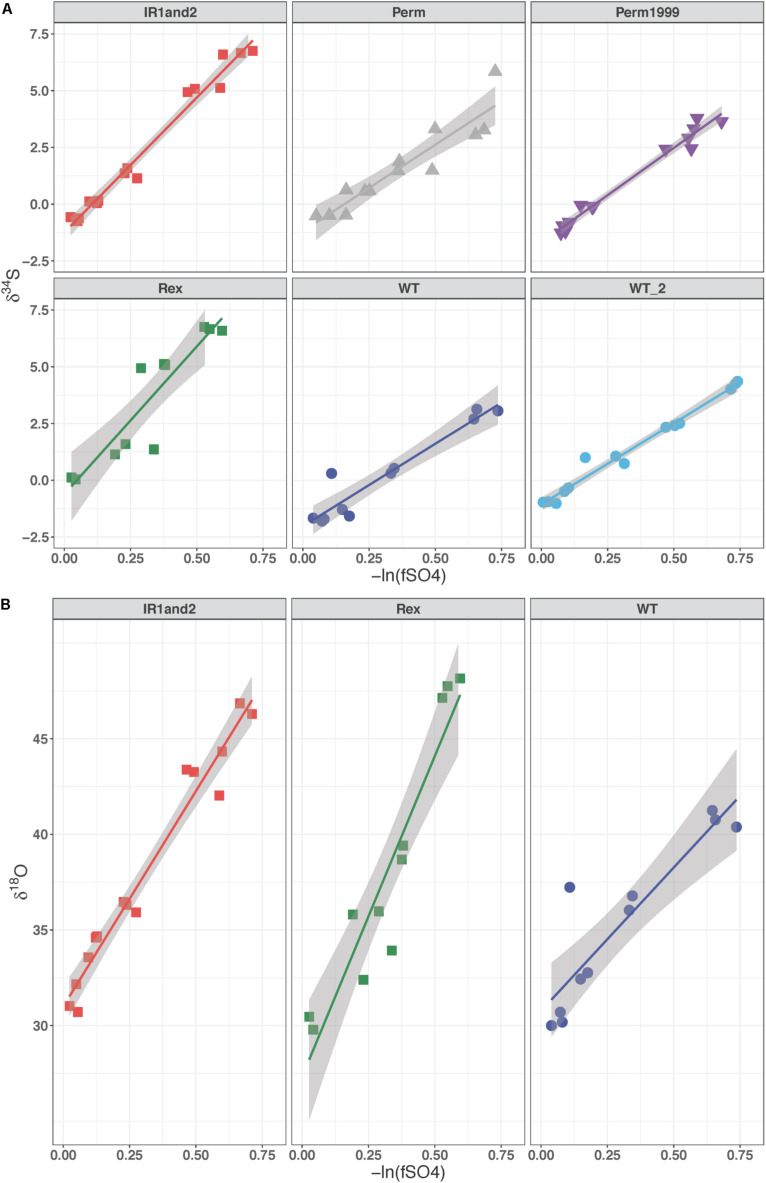
**(A)** δ^34^S of residual sulfate plotted against the negative natural logarithm of the fraction of reactant sulfate remaining for the Permease and Rex mutant batch experiments. **(B)** δ^18^O of residual sulfate plotted against the negative natural logarithm of the fraction of reactant sulfate remaining in the Rex mutant experiments. The slope of these lines represents the fractionation factor (ε) and is plotted with the 95% confidence interval for the regression.

For all batch experiments, sulfate δ^34^S values increased as growth progressed, with an increase up to 8‰ (Figure 4A). The permease mutants, ΔPerm and Perm1999, exhibited ∼1‰ larger ^34^ε fractionations than the WT strain (Table 2 and Figure 4A); however, this difference is small and approaches the uncertainties of the determinations. The Rex-related mutant strains Δ*rex* and IR1and2 both showed larger ^34^ε than the WT by ∼5‰ (Table 2 and Figure 4A) under comparable growth conditions. The sulfur isotope fractionations reported for all strains are comparable to values reported for DvH and other SRB (Mangalo et al., 2007; Turchyn et al., 2010; Sim et al., 2011a, b; Leavitt et al., 2013, 2016).

**FIGURE 5:**
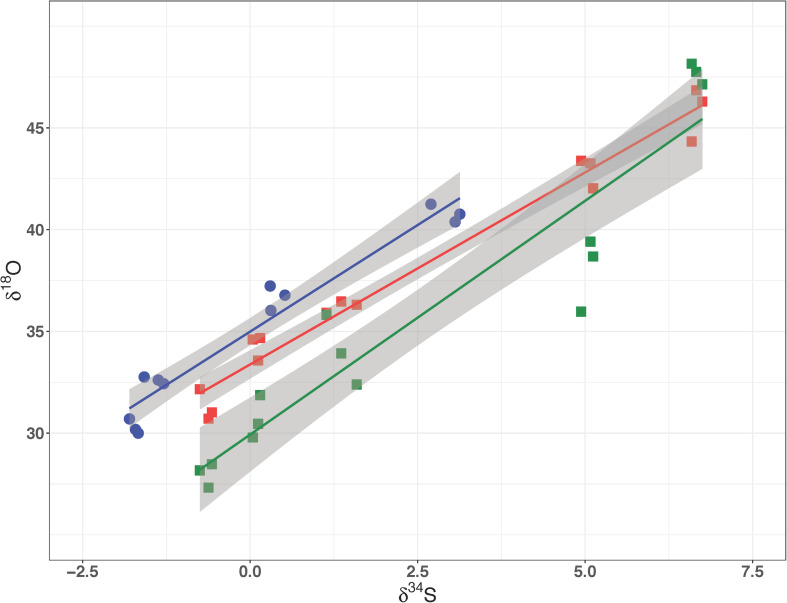
δ^18^O of residual sulfate plotted against its δ^34^S from the Rex batch culture mutant experiment. In all three strains these relationships are linear, suggestive of a dominant kinetic isotope effect. The data for WT, IR1and2, Δ*rex* are in dark blue, red, and green, respectively.

The media water of the Rex experimental set was enriched in ^18^O to a δ^18^O_H2O_ value of +130‰. As growth progressed in the Rex experimental set, residual sulfate became enriched in ^18^O, with δ^18^O_SO__4_ increasing from +29‰ to between +40‰ and +50‰ by the end of growth. The increase in the value of δ^18^O_SO__4_ with reaction progress in each sample is shown in Figure 4B. The values of ^18^ε were larger in both the Δ*rex* and IR1and2 compared to the WT (Table 2). In these experiments, δ^34^S_SO__4_ and δ^18^O_SO__4_ were linearly correlated (Figure 5).

### Sat Expression Affects Isotopic Fractionation

**FIGURE 6:**
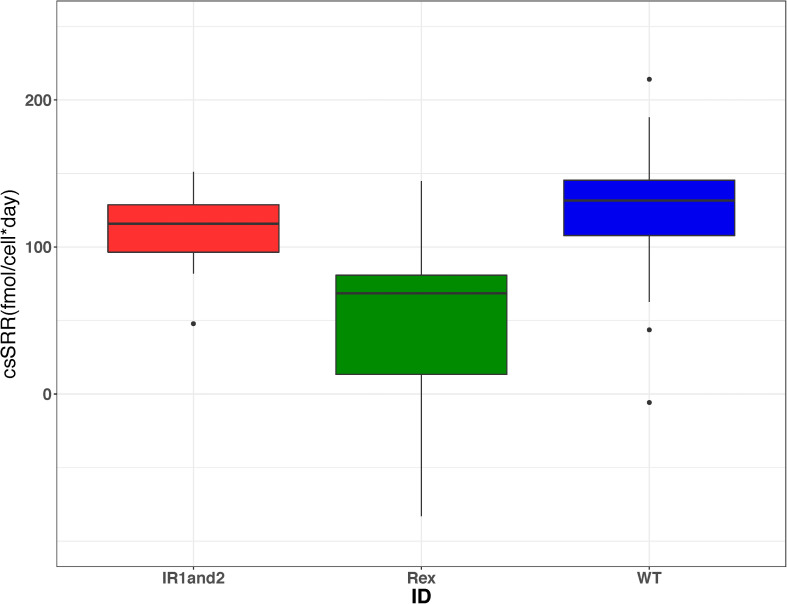
Boxplots showing the range of cell-specific sulfate reduction rates (csSRR) calculated from each harvested bottle in each of the three strains from the Rex batch culture experiments. These results show that the csSRR for the wild type is greater than that in the Δ*rex* strain, but indistinguishable from the IR1and2 strain. The data for WT, IR1and2, Δ*rex* are in dark blue, red, and green, respectively.

**FIGURE 7:**
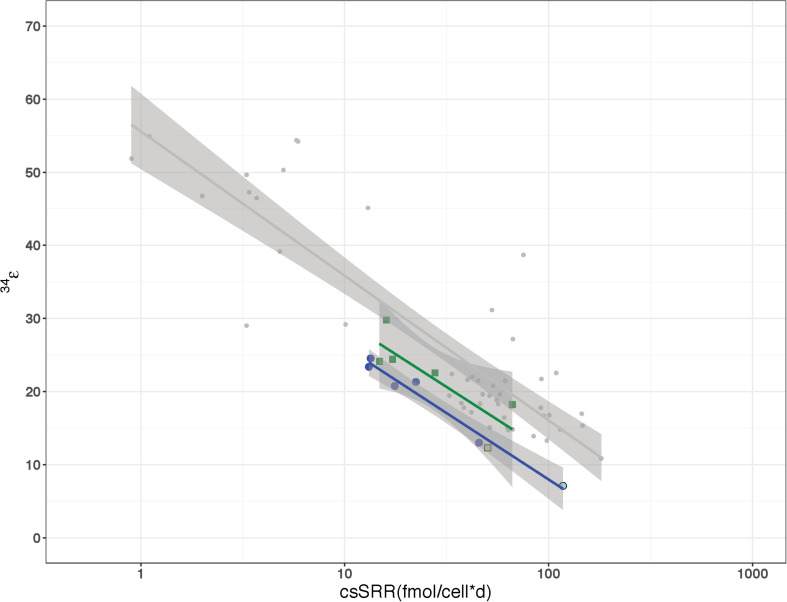
Relationship of cell-specific sulfate reduction rates (csSRR) to sulfur fractionation factor (^34^ε) for *D. vulgaris* as previously reported by Leavitt et al. (2013) in comparison to the wild type strain (dark blue) and Δ*rex* (green) grown in continuous and batch culture. The light blue and green colored symbols represent the batch fractionation factors for WT and Δ*rex*, respectively.

The role of Sat expression in isotope fractionation was investigated. To isolate the isotope effects of this enzyme, alterations in sulfate reduction rate were examined. Previous studies have observed that ^34^ε varies strongly as a function of csSRR (Rees, 1973; Brunner and Bernasconi, 2005; Johnston et al., 2005, 2007; Bradley et al., 2011, 2016; Sim et al., 2011a; Leavitt et al., 2013; Wing and Halevy, 2011). We calculated csSRR in our Rex batch cultures (Figure 6) and compared both csSRR and growth rate to observed fractionations (Figure 7) to determine whether this relationship could account for the observed patterns.

The perturbations of Sat expression in Rex-related mutant strains impacted the observed sulfate oxygen and sulfur isotope fractionations. Although the WT and IR1and2 strains have csSRR that are statistically indistinguishable, the ^18^ε values decrease with increasing csSRR (Table 2). However, in the WT, IR1and2, and Δ*rex* strains neither the relationship of ^18^ε with csSRR (*p* = 0.35) nor the relationship of ^18^ε with growth rate (*p* = 0.24) shows a discernable correlation. The magnitude of ^34^ε for all three strains showed an inverse relationship with growth rate (*p* = 0.045), but no relationship with csSRR (*p* = 0.54) (Table 2).

### Isolation of Growth Rate Effects in Chemostats

Previous work has shown that csSRR is correlated with ^34^ε (Detmers et al., 2001; Sim et al., 2011b; Leavitt et al., 2013). The increased fractionation factors in the Δ*rex* strain over the WT strain in batch could be explained via the decreased csSRR of Δ*rex*. However, the csSRR for the WT and IR1and2 strains were indistinguishable so it is also possible to conclude that perturbed Sat activity was responsible for the differences in fractionations.

In order to clarify the issues of growth rate and csSRR effects on the sulfur isotope fractionation differences between WT and Δ*rex*, these two strains were grown in parallel chemostats where the same steady state growth rate was maintained in each vessel (e.g., two chemostats run simultaneously using the same pump to maintain the same dilution rates). We examined five steady-state rates ranging from 0.008 to 0.051 hr^–1^, which corresponds to a range of 0.096–0.614 of μ_*max*_ for the WT and a range of 0.129–0.823 of μ_max_ for Δ*rex*. The effluent δ^34^S_SO__4_ values of Δ*rex* (16.4–9.4‰) partially overlapped with the more enriched values of the WT (12.8–5.9‰) (*p* = 0.02), relative to the media sulfate that has a δ^34^S of −2.3‰. The evolved δ^34^S_H__2__S_ values of Δ*rex* (−13.2 to −8.5‰) were more depleted than the values of WT (−10.4 to −6.7‰). The ^34^ε of Δ*rex* was slightly larger than that of the WT (*p* = 0.063) (Table 2). If the ^34^ε and csSRR values for both batch and continuous are considered, the slope of the regressions for the Δ*rex* and WT strains are nearly equal to one another (−7.65 and −7.42, respectively) and to the slope (−8.59) that Leavitt et al. (2013) showed previously in the same strain (Figure 7). The only difference between the Δ*rex* and WT regressions, when batch and continuous data were plotted together, were the y-intercepts of 47.0 and 42.1‰, respectively. In both the batch and chemostat cultures, strains with increased Sat transcription differed in ^34^ε by ∼5‰ relative to the wild type.

## Discussion

### Fractionation in Permease Mutants

The two permease mutants, ΔPerm and Perm1999, showed an increase in ^34^ε of ∼1‰ relative to the wild type strain (WT_2_) (Table 2). Interpretation of these results must consider both the sulfate import mechanism and the change in csSRR, as well as the potential overlaps in uncertainties. The csSRR for ΔPerm is lower than the wild type (*p* = 0.013), and not clearly distinguishable from Perm1999 (*p* = 0.21). The hypothesis tested in this set of experiments was that perturbations in permease expression would change sulfate import rates and lead to altered fractionation. While little change in fractionation was observed, the results are confounded by changes in csSRR.

*Desulfovibrio vulgaris* str. Hildenborough (DvH) is still able to carry out sulfate reduction following the deletion of DVU0053, DVU0279, and DVU1999, which is consistent with the presence of additional mechanisms for sulfate transport (Marietou et al., 2018). The observed difference in ^34^ε may suggest that the remaining transporters have a different fractionation, or that their expression changed in response to the deleted genes. However, given these results, there is little evidence that these particular sulfate transporters have much contribution to the overall expression of ^34^ε.

### Increased ^18^ε and ^34^ε Due to Sat Expression

A linear correlation of δ^18^O_SO__4_ vs. δ^34^S_SO__4_ has been interpreted to imply that the fractionation of oxygen isotopes is controlled by kinetic processes, rather than inorganic equilibrium (Mandernack et al., 2003; Turchyn et al., 2010; Mills et al., 2016; Antler et al., 2017). In contrast, when equilibrium processes dominate, the plot of δ^18^O_SO__4_ vs. δ^34^S_SO__4_ is curved, approaching an asymptote of the value of δ^18^O_SO__4_ that is in equilibrium with water.

The relationships of δ^18^O_SO__4_ vs. δ^34^S_SO__4_ in residual sulfate produced by the wild type *D. vulgaris* is similar to that of Δ*rex* and in IR1and2. All three plots show a linear relationship between oxygen and sulfur isotope ratios with similar slopes. The wild type strain has a higher δ^18^O_SO__4_ intercept than the mutants – i.e., at the point when residual sulfate δ^18^O_SO__4_ has evolved to a similar value in all three strains, the mutant strains have produced residual sulfate that is more enriched in ^34^S. These patterns suggest that in all these experiments, enrichment of ^18^O in residual sulfate is predominantly due to kinetic fractionation. Table 2 suggests that both ^18^ε and ^34^ε are higher in Δ*rex* and in IR1and2 than in the wild type.

Given these results, the question becomes: why does increased transcription of Sat result in higher kinetic ^18^ε and ^34^ε? We will exclude the Δ*rex* result: this strain had a lower csSRR than the wild type, and there is no requirement to attribute the observed changes in fractionation to any mechanism other than the observed change in csSRR. In the chemostat, Δ*rex* showed a slight increase in fractionation relative to the wild type, but it was not clearly distinguishable. However, the csSRR for IR1and2 was similar to the wild type. One hypothesis is that increased Sat transcription results in increased Sat expression, and that this increases the rate at which intracellular sulfate is converted to APS, yielding a higher intracellular APS pool. This affects the expressed fractionation of the downstream steps. For example, if APS reduction is rate-limiting, then two strains operating at the same csSRR will have approximately equal rates of APS reduction. APS will be less quantitatively reduced in the strain with a higher intracellular pool of APS – and such a strain would be predicted to express higher isotopic fractionations at this step, as we observe in this experiment. In contrast, if sulfite reduction was rate limiting, then we would predict a significant back-flux from sulfite toward sulfate. This back-flux would carry the ^18^O signature of equilibrium exchange between sulfite and water. If we exclude this interpretation based on the linear relationship between δ^18^O_SO__4_ vs. δ^34^S_SO__4_, then we can draw two inferences. First, that APS reduction is likely the rate-limiting step under these growth conditions. This conclusion is in accord with modeled metabolism of this strain, for other experiments grown under similar conditions (Wing and Halevy, 2011). Second, while we have no direct evidence that the IR1and2 strain has increased Sat expression or APS concentrations, these conditions are likely, given the increased Sat transcription in this strain. The inference that follows is that it is possible to change the isotope fractionations expressed during APS reduction by at least 15‰ for ^18^ε and 5‰ ^34^ε, by perturbing upstream fluxes.

Other explanations should also be considered. Cellular overproduction of APS in the IR1and2 mutant is likely to result in hydrolysis of some of that APS (Baddiley et al., 1957; Robbins and Lipmann, 1958) – this would form sulfate from APS but without the concomitant recovery of ATP. This condition would exist, and even be exacerbated in the Δ*rex* mutant, where a wider loss of transcriptional regulation may perturb cellular homeostasis. The effects of this on isotopic fractionation are difficult to predict, but given the myriad interactions between the enzymes of DSR and the range of electron carriers in the cell, it is not surprising to observe differences from the wild type. Rex may not be the only mechanism regulating Sat expression. The presence of APS has been shown to inhibit the activity of Sat in both the dissimilatory and assimilatory versions of the enzyme across multiple domains of life (Seubert et al., 1983, 1985; Renosto et al., 1984, 1990, 1991; Wang et al., 1995; MacRae et al., 2001; Hanna et al., 2002; Yu et al., 2007; Gay et al., 2009; Parey et al., 2013; Ravilious et al., 2013; Herrmann et al., 2014). Increased Sat transcription therefore may not translate linearly to increased Sat activity, particularly if it results in increased APS accumulation.

## Conclusion

Mutant strains of the sulfate reducing bacterium *D. vulgaris* str. Hildenborough with mutations deleting sulfate transporters showed only small changes in overall sulfur isotope fractionation. The mutations targeted all the sulfate transporters known at the time they were constructed, but the continued ability of these strains to reduce sulfate, along with more recent genomic work, suggests that these strains have other sulfate transporters. More detailed analysis of the full range of sulfate transporters would be required to determine whether the sulfate affinity of individual transporters affects the overall expressed fractionation of the strains under various conditions.

Mutant strains targeting the expression of Sat showed differences in ^18^ε and ^34^ε relative to wild type. In the case of the Δ*rex* strain, we cannot conclude that there were any differences in fractionation apart from those imposed by a growth defect, or that differences were solely attributable to differential Sat expression. In the IR1and2 strain, however, increased ^18^ε and ^34^ε probably results from increased Sat expression. The linearity of the relationship between residual sulfate δ^18^O_SO__4_ vs. δ^34^S_SO__4_ suggests that these strains showed increased kinetic fractionation. These patterns are consistent with APS reduction as a rate-limiting step for sulfate reduction in this strain under these conditions. This demonstrates that variation in expressed fractionation can be imposed by perturbing fluxes in the portion of the metabolic network upstream from APS reduction, and places a minimum magnitude on the variation in fractionations that can be imposed at this step.

## Data Availability Statement

All datasets generated for this study are included in the article/Supplementary Material.

## Author Contributions

DS, DJ, and AB designed the project and experiments. DS conducted the experiments. DS, DF, and DJ conducted isotope analyses. DS, DF, DJ, and AB analyzed the isotope data. DS and AB analyzed the geochemical and growth data. All authors contributed to the manuscript writing.

## Conflict of Interest

The authors declare that the research was conducted in the absence of any commercial or financial relationships that could be construed as a potential conflict of interest.

## References

[B1] Antler G. Turchyn A. V. Ono S. Sivan O. Bosak T. (2017).. Combined 34S, 33S and 18O isotope fractionation record different intracellular steps of microbial sulfate reduction. *Geochim. Cosmochim. Acta.* 203 364–380 10.1016/j.gca.2017.01.015

[B2] Antler G. Turchyn A. V. Rennie V. Herut B. Sivan O. (2013). Coupled sulfur and oxygen isotope insight into bacterial sulfate reduction in the natural environment. *Geochim. Cosmochim. Acta.* 118 98–117. 10.1016/j.gca.2013.05.005

[B3] Baddiley J. Buchanan J. G. Letters R. (1957). Synthesis of Adenosine-5’ Sulphatophosphate. A degradation product of an intermediate enzymatic synthesis of sulphuric esters. *J. Chem. Soc.* 1957 1067–1071. 10.1039/jr9570001067

[B4] Bao H. (2006). Purifying barite for oxygen isotope measurement by dissolution and reprecipitation in a chelating solution. *Anal. Chem.* 78 304–309. 10.1021/ac051568z 16383341

[B5] Betts R. H. Voss R. H. (1970). The kinetics of oxygen exchange between the sulfite ion and water. *Can. J. Chem.* 48 2035–2041. 10.1139/v70-339

[B6] Bradley A. S. Leavitt W. D. Johnston D. T. (2011). Revisiting the dissimilatory sulfate reduction pathway. *Geobiology* 9 446–457. 10.1111/j.1472-4669.2011.00292.x 21884365

[B7] Bradley A. S. Leavitt W. D. Schmidt M. Knoll A. H. Girguis P. R. Johnston D. T. (2016). Patterns of sulfur isotope fractionation during microbial sulfate reduction. *Geobiology* 14 91–101. 10.1111/gbi.12149 26189479

[B8] Brunner B. Bernasconi S. M. (2005). A revised isotope fractionation model for dissimilatory sulfate reduction in sulfate reducing bacteria. *Geochimi. Cosmochim. Acta* 69 4759–4771. 10.1016/j.gca.2005.04.015

[B9] Brunner B. Bernasconi S. M. Kleikemper J. Schroth M. H. (2005). A model for oxygen and sulfur isotope fractionation in sulfate during bacterial sulfate reduction processes. *Geochimi. Cosmochim. Acta* 69 4773–4785. 10.1016/j.gca.2005.04.017

[B10] Canfield D. E. (2001). Isotope fractionation by natural populations of sulfate-reducing bacteria. *Geochimi. Cosmochim. Acta* 65 1117–1124. 10.1016/s0016-7037(00)00584-6

[B11] Canfield D. E. (2004). The evolution of the earth surface sulfur reservoir. *Am. J. Sci.* 304 839–861. 10.2475/ajs.304.10.839 12377050

[B12] Canfield D. E. Farquhar J. Zerkle A. L. (2010). High isotope fractionations during sulfate reduction in a low-sulfate euxinic ocean analog. *Geology* 38 415–418. 10.1130/g30723.1

[B13] Chiba H. Sakai H. (1985). Oxygen isotope exchange rate between dissolved sulfate and water at hydrothermal temperatures. *Geochim. Cosmochim. Acta* 49 993–1000. 10.1016/0016-7037(85)90314-x

[B14] Christensen G. A. Zane G. M. Kazakov A. E. Li X. Rodlonov D. A. Novichkov P. S. (2015). Rex (Encoded by DVU_0916) in *Desulfovibrio vulgaris* Hildenborough is a repressor of sulfate adenylyl transferase and is regulated by NADH. *J. Bacteriol.* 197 29–39. 10.1128/jb.02083-14 25313388 PMC4288696

[B15] Clark M. E. He Q. Huang K. H. Alm E. J. Wan X. F. Hazen T. C. (2006). Temporal transcriptomic analysis of *Desulfovibrio vulgari*s Hildenborough transitions into stationary phase during electron donor depletion. *Appl. Environ. Microbiol.* 72 5578–5588. 10.1128/aem.00284-06 16885312 PMC1538716

[B16] Cline J. D. (1969). Spectrophotometric determination of hydrogen sulfide in natural water. *Limnol. Ocean* 14 454–458. 10.4319/lo.1969.14.3.0454

[B17] Detmers J. Brüchert V. Habicht K. S. Kuever J. (2001). Diversity of sulfur isotope fractionations by sulfate-reducing prokaryotes. *Appl. Environ. Microbiol.* 67 888–894. 10.1128/aem.67.2.888-894.2001 11157259 PMC92663

[B18] Farquhar J. Canfield D. E. Masterson A. Bao H. Johnston D. T. (2008). Sulfur and oxygen isotope study of sulfate reduction in experiments with natural populations from Fællestrand, Denmark. *Geochim*. *Cosmochim. Acta* 72 2805–2821. 10.1016/j.gca.2008.03.013

[B19] Farquhar J. Johnston D. T. Wing B. A. Habicht K. S. Canfield D. E. Airieau S. (2003). Multiple sulphur isotopic interpretations of biosynthetic pathways: implications for biological signatures in the sulphur isotope record. *Geobiology.* 1 27–36. 10.1046/j.1472-4669.2003.00007.x

[B20] Fike D. A. Bradley A. S. Rose C. V. (2015). Rethinking the ancient sulfur cycle. *Annu. Rev. Earth Planet. Sci.* 43 593–622. 10.1146/annurev-earth-060313-054802

[B21] Fritz P. Basharmal G. M. Drimmie R. J. Ibsen J. Qureshi R. M. (1989). Oxygen isotope exchange between sulphate and water during bacterial reduction of sulphate. *Chem. Geol.* 79 99–105. 10.1016/0168-9622(89)90012-2

[B22] Gay S. C. Fribourgh J. L. Donohue P. D. Segel I. H. Fisher A. J. (2009). Kinetic properties of ATP Sulfurylase and APS kinase from *Thiobacillus denitrificans*. *Arch. Biochem. Biophys.* 489 110–117. 10.1016/j.abb.2009.07.026 19664586

[B23] Gyan S. Shiohira Y. Sato I. Takeuchi M. Sato T. (2006). Regulatory loop between redox sensing of the NADH/NAD+ ratio by Rex (YdiH) and oxidation of NADH by NADH dehydrogenase Ndh in *Bacillus subtilis*. *J. Bacteriol.* 188 7062–7071. 10.1128/jb.00601-06 17015645 PMC1636230

[B24] Habicht K. S. Canfield D. E. (1997). Sulfur isotope fractionation during bacterial sulfate reduction in organic-rich sediments. *Geochim. Cosmochim. Acta* 61 5351–5361. 10.1016/s0016-7037(97)00311-6 11541664

[B25] Hanna E. MacRae I. J. Medina D. C. Fisher A. J. Segel I. H. (2002). ATP sulfurylase from the hyperthermophilic chemolithotroph *Aquifex aeolicus*. *Arch. Biochem. Biophys.* 406 275–288. 10.1016/s0003-9861(02)00428-9 12361716

[B26] Hayes J. M. (1983).). “Practice and principles of isotopic measurements in organic geochemistry,” in *Organic Geochemistry of Contemporaneous and Ancient Sediments*, ed. Meinschein W. G. (Bloomington, IN: Society of Economic Paleontologists and Mineralogists), 5.1–5.31.

[B27] Hayes J. M. (2001). Fractionation of carbon and hydrogen isotopes in biosynthetic processes. *Rev. Mineral. Geochem.* 43 225–277. 10.1515/9781501508745-006

[B28] Herrmann J. Ravilious G. E. McKinney S. E. Westfall C. S. Lee S. G. Baraniecka P. (2014). Structure and mechanism of soybean ATP Sulfurylase and the committed step in plant sulfur assimilation. *J. Biol. Chem.* 289 10919–10929. 10.1074/jbc.m113.540401 24584934 PMC4036203

[B29] Johnston D. T. Farquhar J. Canfield D. E. (2007). Sulfur isotope insights into microbial sulfate reduction: when microbes meet models. *Geochim. Cosmochim. Acta* 71 3929–3947. 10.1016/j.gca.2007.05.008

[B30] Johnston D. T. Farquhar J. Wing B. A. Kaufman A. J. Canfield D. E. Habicht K. S. (2005). Multiple sulfur isotope fractionations in biological systems: a case study with sulfate reducers and sulfur disproportionators. *Am. J. Sci.* 305 645–660. 10.2475/ajs.305.6-8.645 12377050

[B31] Jørgensen B. B. (1982). Mineralization of organic-matter in the sea bed - the role of sulfate reduction. *Nature.* 296 643–645. 10.1038/296643a0

[B32] Keller K. L. Bender K. S. Wall J. D. (2009). Development of a markerless genetic exchange system for *Desulfovibrio vulgaris* Hildenborough and its use in generating a strain with increased transformation efficiency. *Appl. Environ. Microbiol.* 75 7682–7691. 10.1128/aem.01839-09 19837844 PMC2794091

[B33] Kolter R. Siegele D. A. Tormo A. (1993). The stationary phase of the bacterial life cycle. *Annu. Rev. Microbiol* 47 855–874. 10.1146/annurev.mi.47.100193.004231 8257118

[B34] Korte H. L. Fels S. R. Christensen G. A. Price M. N. Kuehl J. V. Zane G. M. (2014). Genetic basis for nitrate resistance in *Desulfovibrio* strains. *Front. Microbiol.* 5:153. 10.3389/fmicb.2014.00153 24795702 PMC4001038

[B35] Kuehl J. V. Price M. N. Ray J. Wetmore K. M. Esquivel Z. Kazakov A. E. (2014). Functional genomics with a comprehensive library of transposon mutants for the sulfate-reducing bacterium *Desulfovibrio alaskensis* G20. *mBio* 5 1–13. 10.1128/mBio.01041-14 PMC4045070 24865553

[B36] Leavitt W. D. Bradley A. S. Santos I. A. C. Johnston D. T. (2015). Sulfur isotope effects of dissimilatory sulfite reductase. *Front. Microbiol.* 6:1392. 10.3389/fmicb.2015.01392 26733949 PMC4690157

[B37] Leavitt W. D. Halevy I. Bradley A. S. Johnston D. T. (2013). Influence of sulfate reduction rates on the Phanerozoic sulfur isotope record. *PNAS* 110 11244–11249. 10.1073/pnas.1218874110 23733944 PMC3710818

[B38] Leavitt W. D. Venceslau S. Santos I. A. C. Johnston D. T. Bradley A. S. (2016). Fractionation of sulfur and hydrogen isotopes in *Desulfovibrio vulgaris* str. *Hildenborough with perturbed DsrC expression*. *FEMS Microbiol. Lett.* 363:fnw226. 10.1093/femsle/fnw226 27702753

[B39] Lloyd R. M. (1968). Oxygen isotope behavior in the sulfate-water system. *J. Geophys. Res.* 73 6099–6110. 10.1029/jb073i018p06099

[B40] MacRae I. J. Segel I. H. Fisher A. J. (2001). Crystal structure of ATP Sulfurylase from *Penicillium chrysogenum*: insights into the allosteric regulation of sulfate assimilation. *Biochem.* 40 6795–6804. 10.1021/bi010367w 11389593

[B41] Mandernack K. W. Krouse H. R. Skei J. M. (2003). A stable sulfur and oxygen isotopic investigation of sulfur cycling in an anoxic marine basin, Framvaren, Fjord, Norway. *Chem. Geol.* 195 181–200. 10.1016/s0009-2541(02)00394-7

[B42] Mangalo M. Meckenstock R. U. Stichler W. Ensiedl F. (2007). Stable isotope fractionation during bacterial sulfate reduction is controlled by reoxidation of intermediates. *Geochim. Cosmochim. Acta* 71 4161–4171. 10.1016/j.gca.2007.06.058

[B43] Marietou A. Røy H. Jørgensen B. B. Kjeldsen K. U. (2018). Sulfate transporters in dissimilatory sulfate reducing microorganisms: a comparative genomic analysis. *Front. Microbiol.* 9:309. 10.3389/fmicb.2018.00309 29551997 PMC5840216

[B44] Mills J. V. Antler G. Turchyn A. V. (2016). Geochemical evidence for cryptic sulfur cycling in salt marsh sediments. *Earth Planet. Sci. Lett.* 453 23–32. 10.1016/j.epsl.2016.08.001

[B45] Navarro Llorens J. M. Tormo A. Martínez-García E. (2010). Stationary phase in gram-negative bacteria. *FEMS Microbiol. Rev.* 34 476–495. 10.1111/j.1574-6976.2010.00213.x 20236330

[B46] Parey K. Demmer U. Warkentin E. Wynen A. ErmLer U. Dahl C. (2013). Structural, biochemical, and genetic characterization of dissimilatory ATP sulfurylase from *Allochromatium vinosum*. *PLoS One* 8:e74707. 10.1371/journal.pone.0074707 24073218 PMC3779200

[B47] Price M. N. Wetmore K. M. Waters R. J. Callaghan M. Ray J. Liu H. (2018). Mutant phenotypes for thousands of bacterial genes of unknown function. *Nature* 557 503–509. 10.1038/s41586-018-0124-0 29769716

[B48] Rabus R. Hansen T. A. Widdel F. (2013). *“Dissimilatory sulfate- and sulfur-reducing Prokaryotes,” in The Prokaryotes - Prokaryotic Physiology and Biochemistry, eds E. Rosenberg et al.* Berlin, Germany: Springer-Verlag, 310–404.

[B49] Ravcheev D. A. Li X. Latif H. Zengler K. Leyn S. A. Korostelev Y. D. (2012). Transcriptional regulation of central carbon and energy metabolism in bacteria by redox-responsive repressor Rex. *J. Bacteriol.* 194 1145–1157. 10.1128/jb.06412-11 22210771 PMC3294762

[B50] Ravilious G. E. Herrmann J. Lee S. G. Westfall C. S. Jez J. J. (2013). Kinetic mechanism of the dimeric ATP Sulfurylase from plants. *Biosci. Rep.* 33 585–591. 10.1042/BSR20130073 PMC3728988 23789618

[B51] Rees C. E. (1973). A steady-state model for sulphur isotope fractionation in bacterial reduction processes. *Geochim. Cosmochim. Acta* 37 1141–1162. 10.1016/0016-7037(73)90052-5

[B52] Renosto F. Martin R. L. Borrell J. L. Nelson D. C. Segel I. H. (1991). ATP Sulfurylase from trophosome tissue of *Riftia pachyptila* (hydrothermal vent tube worm). *Arch. Biochem. Biophys.* 290 66–78. 10.1016/0003-9861(91)90592-7 1898101

[B53] Renosto F. Martin R. L. Wailes L. M. Daley L. A. Segel I. H. (1990). Regulation of inorganic sulfate activation in filamentous fungi. *J. Biol. Chem.* 265 10300–10308. 2162344

[B54] Renosto F. Seubert P. A. Segel I. H. (1984). Adenosine 5’-phosphosulfate kinase from *Penicillium chrysogenum*. *J. Biol. Chem.* 259 2113–2123. 6321459

[B55] Robbins P. W. Lipmann F. (1958). Separation of the two enzymatic phases in active sulfate synthesis. *J. Biol. Chem.* 233 681–685. 13575436

[B56] Rudnicki M. D. Elderfield H. Spiro B. (2001). Fractionation of sulfur isotopes during bacterial sulfate reduction in deep ocean sediments at elevated temperatures. *Geochim. Cosmochim. Acta* 65 777–789. 10.1016/s0016-7037(00)00579-2

[B57] Santos A. S. Venceslau S. S. Grein F. Leavitt W. D. Dahl C. Johnston D. T. (2015). A protein trisulfide couples dissimilatory sulfate reduction to energy conservation. *Science* 350 1541–1545. 10.1126/science.aad3558 26680199

[B58] Seubert P. A. Hoang L. Renosto F. Segel I. H. (1983). ATP Sulfurylase from *Penicillium chrysogenum*: measurements of the true specific activity of an enzyme subject to the potent product inhibition and a reassessment of the kinetic mechanism. *Arch. Biochem. Biophys.* 225 679–691. 10.1016/0003-9861(83)90079-6 6312889

[B59] Seubert P. A. Renosto F. Knudson P. Segel I. H. (1985). Adenosinetriphosphate Sulfurylase from *Penicillium chrysogenum*: steady-state kinetics of the forward and reverse reactions, alternative substrate kinetics, and equilibrium binding studies. *Arch. Biochem. Biophys.* 240 509–523. 10.1016/0003-9861(85)90057-8 2992379

[B60] Shen Y. Buick R. Canfield D. E. (2001). Isotopic evidence for microbial sulphate reduction in the early Archaean era. *Nature.* 410 77–81. 10.1038/35065071 11242044

[B61] Sim M. S. Bosak T. Ono S. (2011a). Large sulfur isotope fractionation does not require disproportionation. *Science* 1 74–77. 10.1126/science.1205103 21719675

[B62] Sim M. S. Ogata H. Lubitz W. Adkins J. F. Sessions A. L. Orphan V. J. (2019). Role of APS reductase in biogeochemical sulfur isotope fractionation. *Nat. Commun.* 10:44. 10.1038/s41467-018-07878-4 PMC6327049 30626879

[B63] Sim M. S. Ono S. Donovan K. Templer S. P. Bosak T. (2011b). Effect of electron donors on the fractionation of sulfur isotopes by a marine *Desulfovibrio* sp. *Geochim. Cosmochim. Acta* 75 4244–4259. 10.1016/j.gca.2011.05.021

[B64] Thode G. E. Kleerekoper H. McElcheran D. E. (1951). Isotope fractionation in the bacterial reduction of sulphate. *Res. London.* 4 581–582.

[B65] Turchyn A. V. Brüchert V. Lyons T. W. Engel G. S. Balci N. Schrag D. P. (2010). Kinetic oxygen isotope effects during dissimilatory sulfate reduction: a combined theoretical and experimental approach. *Geochim. Cosmochim. Acta* 74 2011–2024. 10.1016/j.gca.2010.01.004

[B66] Wang R. Changxian L. Leyh T. S. (1995). Allosteric regulation of the ATP Sulfurylase associated GTPase. *Biochemistry* 34 490–495. 10.1021/bi00002a013 7819241

[B67] Wankel S. D. Bradley A. S. Eldridge D. L. Johnston D. T. (2014). Determination and application of the equilibrium oxygen isotope effect between water and sulfite. *Geochim. Cosmochim. Acta* 125 694–711. 10.1016/j.gca.2013.08.039

[B68] Wing B. A. Halevy I. (2011). Intracellular metabolite levels shape sulfur isotope fractionation during microbial sulfate respiration. *PNAS* 111 18116–18125. 10.1073/pnas.1407502111 25362045 PMC4280625

[B69] Wortmann U. G. Bernasconi S. M. Böttcher M. E. (2001). Hypersulfidic deep biosphere indicates extreme sulfur isotope fractionation during single-step microbial sulfate reduction. *Geology* 29 647–650. 10.1130/0091-7613(2001)029<0647:hdbies>2.0.co;2

[B70] Yu Z. Landson E. B. Segel I. H. Fisher A. J. (2007). Crystal structure of the bifunctional ATP Sulfurylase - APS kinase from the chemolithotrophic thermophile *Aquifex aeolicus*. *J. Mol. Biol.* 365 732–743. 10.1016/j.jmb.2006.10.035 17095009

[B71] Zane G. M. Yen H. B. Wall J. D. (2010). Effect of the deletion of *qmoABC* and the the promoter-distal gene encoding a hypothetical protein on sulfate reduction in *Desulfovibrio vulgaris* Hildenborough. *Appl. Environ. Micro.* 76 5500–5509. 10.1128/aem.00691-10 20581180 PMC2918943

